# Effects of education level on hypertension, diabetes, and Dyslipidemia among residents in Hezhang County

**DOI:** 10.1016/j.pmedr.2025.103369

**Published:** 2025-12-30

**Authors:** Tang Bin, Jia Xin, Zhu Siqi, Fu Meng, G.U. Yang, Rao Songli, Zhang Ying, Lai Guihong, Zhu Yuqing

**Affiliations:** a International Department of China-Japan Friendship Hospital, 100000 Beijing, China; b Chinese Red Cross Foundation, 100000, Beijing, China; c Hezhang County People's Hospital, 551700 Bijie, Guizhou, China

**Keywords:** Hypertension, Diabetes mellitus, Dyslipidemia-educational attainmen, LDL/HDL-cholesterolemia, Hypertriglyceridemia

## Abstract

**Objective:**

To investigate the prevalence of hypertension, diabetes, and dyslipidemia among residents of Hezhang County and analyze the impact of educational attainment on these conditions.

**Methods:**

A multi-stage cluster random sampling method was used to select 10,000 permanent residents aged ≥18 years in Hezhang County from October to December 2023. Multivariate logistic regression was employed to evaluate the association between educational level and hypertension, diabetes, and dyslipidemia. Mediation analysis was conducted to explore the mediating roles of chronic disease risk factors and knowledge of healthy lifestyle-related factors.

**Results:**

Higher education was independently associated with lower risks of hypertension, diabetes, hypercholesterolemia, and hypertriglyceridemia (aOR = 0.88) (*P* < 0.01). No association was found with LDL or HDL abnormalities. Mediation analysis revealed that cognitive status and BMI mediated the effect on hypertension (26.9 % of total effect) and hypertriglyceridemia (73.8 %), while BMI and alcohol use mediated the effect on diabetes (17.0 %).

**Conclusion:**

The level of education is significantly negatively correlated with the risks of hypertension, diabetes and dyslipidemia. These findings emphasize the importance of education in the prevention of chronic diseases and highlight the potential pathways for targeted intervention measures.

## Highlights

• Education negatively associated with the detection rates of chronic conditions.
• Protective effect of education is disease-specific.
• Cognitive status, lifestyle mediate education's protective effect.

## 1 Introduction

Non-communicable diseases (NCDs), particularly cardiometabolic disorders such as hypertension, diabetes, and dyslipidemia, have emerged as a major global public health challenge due to their high prevalence, long-term disability burden, and socioeconomic impact(Adhra et al., 2025). As the world's most populous nation, China faces an especially heavy NCD burden (Zhixin et al., 2024). According to the Report on Nutrition and Chronic Disease Status of Chinese Residents (2020), the prevalence of hypertension among adults aged 18 and older is 27.5 %, diabetes affects 11.9 %, and dyslipidemia reaches 40.4 % nationally(Hui, 2022). These conditions are not only key risk factors for cardiovascular and cerebrovascular events but also contribute to severe complications such as chronic kidney disease and retinopathy, significantly impairing quality of life and straining healthcare resources (Ikramulhaq et al., 2025). Notably, chronic disease distribution exhibits marked socioeconomic gradients, with higher risks observed in low-income, low-education populations (Chen et al., 2022). However, research remains scarce on how educational attainment influences chronic disease development through modifiable behavioral or physiological pathways, particularly in rural Southwest China(Wenli et al., 2015). This study investigates the association between educational attainment and hypertension, diabetes, and dyslipidemia in Hezhang County, Guizhou Province, aiming to elucidate underlying mechanisms and inform evidence-based strategies for grassroots NCD prevention.

This study targets permanent residents aged ≥18 in Hezhang County to address three questions: (1) Does a dose-response relationship exist between educational attainment and hypertension, diabetes, or dyslipidemia (2) Do health knowledge, BMI, smoking, and alcohol consumption mediate the education-chronic disease association (3) Do these mediating effects vary by disease By employing multistage cluster random sampling and analyzing data through multivariate logistic regression and structural equation modeling (SEM), this research aims to fill gaps in understanding SDH-driven NCD disparities in Southwest China while guiding precision interventions. For instance, if health knowledge emerges as a key mediator, grassroots health agencies could collaborate with educational departments to implement community health campaigns; if economic pathways dominate, rural industrial upgrades and healthcare policy reforms may be prioritized.

## 2 Methods

### 2.1 Study design and population

The study targeted permanent residents aged ≥18 years in Hezhang County. This study adopted a cross-sectional design, and the data were surveyed and entered from October 2023 to December 2023. Permanent residents were defined as Chinese citizens who had resided in the area for ≥6 months within the 12 months preceding the survey, including local registered populations but excluding those who had been away for >6 months, non-local residents living in the area for ≥6 months, and individuals residing in functional zones.

Multistage cluster random sampling method was employed: Hezhang County was divided into 30 primary sampling units (5 subdistricts, 10 towns, 3 townships, and 12 ethnic townships). Eight primary units were randomly selected using simple random sampling. From each selected primary unit, three secondary sampling units (communities/villages) were selected via systematic sampling based on population size ranking. Within each selected community/village, 100 households were randomly chosen. All adults aged ≥18 years in these households were enrolled. A “household” was defined as a group of related individuals living together, with at least one permanent resident.

### 2.2 Data collection

The survey included three components: questionnaires, physical measurements, and laboratory tests.Trained investigators conducted face-to-face interviews using standardized questionnaires covering:Demographics: Sex, age, ethnicity, educational attainment, occupation. Behavioral factors: Smoking, alcohol consumption.Medical history: Self-reported diagnoses of hypertension, diabetes, or dyslipidemia.Chronic disease knowledge: A 12-item assessment of risk factor and healthy lifestyle knowledge (1 point per correct answer; total scores categorized as *high* [9–12], *moderate* [5–8], or *low* [0–4]).

Physical Measurements:Anthropometrics:Height, weight, waist circumference, hip circumference.

Blood pressure and heart rate: Measured using calibrated devices. Laboratory Tests: Fasting venous blood samples were collected by trained medical staff for analysis of:Fasting plasma glucose (FPG). Lipid profiles: Total cholesterol (TC), triglycerides (TG), high-density lipoprotein cholesterol (HDL—C), low-density lipoprotein cholesterol (LDL-C).

### 2.3 Diagnostic criteria

Hypertension/suspected hypertension: Systolic blood pressure (SBP) ≥ 140 mmHg, diastolic blood pressure (DBP) ≥90 mmHg, or self-reported diagnosis. Diabetes/suspected diabetes: FPG ≥ 7.0 mmol/L or self-reported diagnosis. Dyslipidemia:Hypercholesterolemia: TC ≥ 6.2 mmol/L. Hypertriglyceridemia: TG ≥ 2.3 mmol/L. High LDL-C: LDL-C ≥ 4.1 mmol/L. Abnormal HDL—C: HDL-C < 1.0 mmol/L. BMI categories:Obesity: BMI ≥28 kg/m^2^. Overweight: 24 ≤ BMI <28 kg/m^2^.Normal: BMI <24 kg/m^2^.

### 2.4 Statistical analysis

Database management: Data were double-entered and validated using Epidata 3.1.Descriptive analysis: Prevalence rates were compared across groups using chi-square tests (SPSS 21.0). Two models were constructed to assess the association between educational attainment and chronic diseases:Model 1: Adjusted for sex, age, ethnicity, and occupation.Model 2: Further adjusted for BMI, smoking, and alcohol use.Mediation analysis: Bootstrap resampling (5000 iterations) tested the mediating roles of health knowledge, BMI, smoking, and alcohol use in the education-chronic disease relationship. Statistical significance was set at *p* < 0.05.

## 3 Results

### 3.1 Detection rates of hypertension, diabetes, and Dyslipidemia in the study population

A total of 10,000 participants were included in this study, with 4261 (42.6 %) being male. Among them: Hypertension or suspected hypertension was identified in 3466 participants (34.7 %). Factors such as gender, age, ethnicity, education level, occupation, overweight/obesity, smoking, alcohol consumption, and cognitive status significantly influenced the detection rate of hypertension. Diabetes or suspected diabetes was identified in 1578 participants (15.8 %). All aforementioned factors except ethnicity influenced the detection rate of diabetes. Hypercholesterolemia was diagnosed in 1150 participants (11.5 %). All factors except ethnicity, smoking, and alcohol consumption affected its detection rate. Hypertriglyceridemia was observed in 3672 participants (36.7 %). All factors except alcohol consumption showed significant associations. High LDL cholesterolemia was detected in 1537 participants (15.4 %), with gender, age, occupation, overweight/obesity, alcohol consumption, and cognitive status being influencing factors. Abnormal HDL levels were found in 1087 participants (10.9 %), linked to gender, education level, occupation, BMI, waist circumference, smoking, and alcohol consumption. Detailed results are summarized in Table 1.

**Table 1:** Characteristics of the study subjects and detection of hypertension, diabetes, and dyslipidemia(*n* = 10,000), recorded by Zhang Ying and Lai Guihong, from October to December 2023.

Variates	Total	Hypertension		Diabetes		High cholesterol		hypertriglyceridemia		High LDL		Low HDL	
		Count(%)	P	Count(%)	P	Count(%)	P	Count(%)	P	Count(%)	P	Count(%)	P
**Sex**													
Male	4261	1635 (38.4)	<0.01	727 (17.1)	0.01	502 (11.8)	0.45	1697 (39.8)	<0.01	764 (17.9)	<0.01	532 (12.5)	<0.01
Female	5739	1831 (31.9)		851 (14.8)		648 (11.3)		1975 (34.4)		773 (13.5)		555 (9.7)	
**Age**													
18–40	3422	359 (10.5)	<0.01	272 (7.9)	<0.01	209 (6.1)	<0.01	941 (27.5)	<0.01	458 (13.4)	<0.01	344 (10.1)	0.08
41–65	4415	1796 (40.7)		871 (19.7)		587 (13.3)		1965 (44.5)		836 (18.9)		484 (11.0)	
Over 65	2163	1311 (60.6)		435 (20.1)		354 (16.4)		766 (35.4)		243 (11.2)		259 (12.0)	
**Nationality**													
Han	9308	3213 (34.5)	<0.01	1484 (15.9)	0.09	1059 (11.4)	0.20	3420 (36.7)	<0.01	1419 (15.2)	0.45	1015 (10.9)	0.24
Yi	330	150 (45.5)		52 (15.8)		48 (14.5)		146 (44.2)		56 (17.0)		41 (12.4)	
others	362	103 (28.5)		42 (11.6)		43 (11.9)		106 (29.3)		62 (17.1)		31 (8.6)	
**Education**													
Primary	4829	2412 (49.9)	<0.01	1001 (20.7)	<0.01	723 (15.0)	<0.01	2053 (42.5)	<0.01	754 (15.6)	0.11	543 (11.2)	0.14
Middle	1772	445 (25.1)		248 (14.0)		179 (10.1)		598 (33.7)		248 (14.0)		191 (10.8)	
High school	893	184 (20.6)		108 (12.1)		70 (7.8)		228 (25.5)		125 (14.0)		77 (8.6)	
College	2506	425 (17.0)		221 (8.8)		178 (7.1)		793 (31.6)		410 (16.4)		276 (11.0)	


Characteristics of the study subjects and detection of hypertension, diabetes, and dyslipidemia,recorded by Zhang Ying and Lai Guihong, from October to December 2023.													
Variates	Total	Hypertension		Diabetes		High cholesterol		hypertriglyceridemia		High LDL		Low HDL	
		Count(%)	P	Count(%)	P	Count(%)	P	Count(%)	P	Count(%)	P	Count(%)	P
**Job**													
Peasantry	5131	2208 (43.0)	<0.01	955 (18.6)	<0.01	703 (13.7)	<0.01	2051 (40.0)	<0.01	866 (16.9)	<0.01	487 (9.5)	<0.01
Government	2118	394 (18.6)		217 (10.2)		150 (7.1)		746 (35.2)		378 (17.8)		260 (12.3)	
Retirement	173	108 (62.4)		54 (31.2)		25 (14.5)		66 (38.2)		27 (15.6)		29 (16.8)	
Unemployed	1746	606 (34.7)		293 (16.8)		194 (11.1)		659 (37.7)		228 (13.1)		250 (14.3)	
Other	832	150 (18.0)		59 (7.1)		78 (9.4)		150 (18)		38 (4.6)		61 (7.3)	
**BMI**													
Normal	4926	1190 (24.2)	<0.01	564 (11.4)	<0.01	493 (10)	<0.01	1467 (29.8)	<0.01	634 (12.9)	<0.01	431 (8.7)	<0.01
Over weight	3666	1471 (40.1)		658 (17.9)		458 (12.5)		1481 (40.4)		643 (17.5)		462 (12.6)	
Obese	2408	805 (33.4)		356 (25.3)		199 (14.1)		724 (51.4)		260 (18.5)		194 (13.8)	
**Waistline**													
Normal	5971	1610 (27.0)	<0.01	753 (12.6)	<0.01	637 (10.7)	0.01	1953 (32.7)	<0.01	881 (14.8)	0.04	561 (9.4)	<0.01
Excessive	4029	1856 (46.1)		825 (20.5)		513 (12.7)		1719 (42.7)		656 (16.3)		526 (13.1)	
**Smoke**													
NO	7171	2335 (32.6)	<0.01	1073 (15.0)	0.01	824 (11.5)	0.99	2533 (35.3)	<0.01	1074 (15)	0.12	711 (9.9)	<0.01
Former smoker	591	225 (38.1)		109 (18.4)		68 (11.5)		214 (36.2)		88 (14.9)		97 (16.4)	
Current smoker	2238	906 (40.5)		396 (17.7)		258 (11.5)		925 (41.3)		375 (16.8)		279 (12.5)	
**Drink**													
NO	7374	2485 (33.7)	<0.01	1221 (16.6)	0.01	836 (11.3)	0.14	2675 (36.3)	0.29	1100 (14.9)	0.04	790 (10.7)	0.24
Occasional	2129	764 (35.9)		281 (13.2)		243 (11.4)		811 (38.1)		365 (17.1)		250 (11.7)	
Often	497	217 (43.7)		76 (15.3)		71 (14.3)		186 (37.4)		72 (14.5)		47 (9.5)	
**cognition**													
Low	1663	1376 (82.7)	<0.01	527 (31.7)	<0.01	425 (25.6)	<0.01	1289 (77.5)	<0.01	413 (24.8)	<0.01	391 (23.5)	<0.01
Medium	2785	1443 (51.8)		728 (26.1)		468 (16.8)		1690 (60.7)		757 (27.2)		409 (14.7)	
High	2086	647 (31)		323 (15.5)		257 (12.3)		693 (33.2)		367 (17.6)		287 (13.8)	
**Total**	**10,000**	**3466 (34.7)**		**1578 (15.8)**		**1150 (11.5)**		**3672 (36.7)**		**1537 (15.4)**		**1087 (10.9)**	

### 3.2 Impact of education level on hypertension, diabetes, and Dyslipidemia

Multivariate logistic regression analysis revealed that higher education levels were associated with lower detection rates of hypertension, hyperglycemia, hypercholesterolemia, and hypertriglyceridemia. After adjusting for gender, age, ethnicity, occupation, BMI, smoking, and alcohol consumption, the adjusted odds ratios (ORs) were 0.82 (95 % CI: 0.78，0.86) for hypertension, 0.83 (95 % CI: 0.78，0.88) for diabetes, 0.85 (95 % CI: 0.80，0.91) for hypercholesterolemia, and 0.88 (95 % CI: 0.84，0.92) for hypertriglyceridemia. However, no significant association was observed between education level and the detection rates of high LDL cholesterolemia or abnormal HDL levels (Table 2).

**Table 2:** The impact of educational level on hypertension, diabetes and dyslipidemia among the research subjects,recorded by Zhang Ying and Lai Guihong, from October to December 2023.

Variables	Model 1			Model 2		
	β	aOR	P	β	aOR	P
Hypertension	−0.230	0.79	<0.01	−0.205	0.82	<0.01
Diabetes	−0.231	0.79	<0.01	−0.191	0.83	<0.01
High cholesterol	−0.160	0.85	<0.01	−0.161	0.85	<0.01
hypertriglyceridemia	−0.155	0.86	<0.01	−0.130	0.88	<0.01
High LDL	0.003	1.00	0.91	0.014	1.01	0.60
Low HDL	0.005	1.00	0.87	0.031	1.03	0.34

(Model 1 takes gender, age, occupation and ethnicity as covariates; Model 2, based on Model 1, adds overweight or obesity status, smoking and drinking as covariates.)

Mediation analysis further demonstrated that cognitive status, BMI, smoking, and alcohol consumption acted as mediators in the relationship between education level and these chronic conditions. Specifically: Higher education levels reduced hypertension risk through improved cognitive status and. For diabetes, the protective effect of education was mediated by reduced BMI and alcohol consumption.For hypertriglyceridemia, education exerted its influence via enhanced cognitive status and reduce alcohol consumption (Table 3).

**Table 3:** The mediating roles of cognitive status, BMI, smoking and drinking in the relationships between education level and hypertension, diabetes, and dyslipidemia,recorded by Zhang Ying and Lai Guihong, from October to December 2023.

Variables	Direct effects	Indirect effects				Indirect effect proportion%
		Cognition	BMI	Smoke	Drink	
Hypertension	−0.174 (−0.225, −0.124)	−0.032 (−0.048, −0.016)	−0.035 (−0.045, −0.026)	−0.001 (−0.004, 0.001)	0.004 (0.000, 0.008)	26.90
Diabetes	−0.190 (−0.252, −0.128)	−0.001 (−0.020, 0.018)	−0.022 (−0.030, −0.016)	−0.004 (−0.008, −0.001)	−0.012 (−0.018, −0.006)	17.00
High cholesterol	−0.162 (−0.232, −0.091)	0.001 (−0.021, 0.023)	−0.007 (−0.012, −0.002)	0.003 (0.000, 0.007)	0.006 (0.001, 0.012)	1.82
Hyper-triglyceridemia	−0.042 (−0.087, 0.002)	−0.090 (−0.106, −0.075)	−0.023 (−0.030, −0.016)	−0.002 (−0.005, −0.000)	−0.003 (−0.007, 0.001)	73.75

## 4 Discussion

Our study provides robust evidence that higher educational attainment is significantly associated with a lower prevalence of key metabolic disorders—hypertension, diabetes, and dyslipidemia—among adults in rural Hezhang County, China. The adjusted odds ratios (AOR) ranged from 0.82 to 0.88, indicating that with each incremental increase in education level, the odds of the outcome decreased by an average of 12–18 %. This aligns with global patterns that position education as a fundamental social determinant of health(Collaborators, G.B.D.C.o.D, 2024). Notably, this protective effect persisted after controlling for key demographic and behavioral factors, underscoring education's independent role.

Despite the limitations imposed by the cross-sectional design, the results of the mediation analysis in this study still provided important insights into the pathways through which education exerts its influence, and revealed the mechanisms under specific conditions. For hypertension, the effect was primarily mediated through improved cognitive status and lower BMI. This suggests that education fosters both the health literacy necessary for understanding risk factors (e.g., salt intake) and the agency to maintain a healthy weight, likely via better dietary choices(Wei et al., 2025). For diabetes, the significant mediators were BMI and Alcohol Consumption, highlighting education's role in mitigating two major metabolic and behavioral risks(Wang et al., 2023). The pathway for hypertriglyceridemia again involved Cognitive Status and BMI, consistent with the condition's link to dietary carbohydrates and physical activity(Feng et al., 2024). Conversely, the lack of association between education and LDL-cholesterol or HDL-cholesterol abnormalities suggests these lipid profiles may be more strongly influenced by genetic factors or entrenched local dietary habits (e.g., consumption of preserved meats) that are less easily modified by individual knowledge(Boumenna et al., 2022).

The socio-economic context of Hezhang County renders these findings particularly significant. In a setting with limited healthcare infrastructure and health information access, individual health literacy—enhanced by education—becomes a critical compensatory resource(Stormacq et al., 2023). The strong mediating role of cognitive status implies that educated individuals are better equipped to navigate preventive care and lifestyle modifications despite systemic gaps(Poyhonen et al., 2025). This underscores that in resource-limited rural areas, educational interventions may yield substantial public health returns.

Our results both confirm and contextualize existing literature. The inverse education-disease gradient is consistent with observations from high-income countries, where education links to better health literacy, healthcare utilization, and healthier behaviors(Nair et al., 2022). However, the identified mediators—especially the prominent role of cognitive status and the negligible impact of factors like smoking—may reflect the unique cultural and lifestyle milieu of rural Southwest China. Traditional dietary practices, family-based behavioral norms, and different patterns of social diffusion of health information may modulate the pathways found in Western populations.

This study has several limitations. Firstly, the mediation analysis based on cross-sectional data can reveal the possible paths between variables, but it cannot strictly establish causal relationships or clearly determine the temporal sequence. The educational level, mediator variables (such as cognitive status, BMI), and chronic disease outcomes were measured at the same time point. Therefore, the ‘mediation effect’ observed should be cautiously interpreted as a statistical association pattern rather than a definite causal mechanism. This association may be influenced by unmeasured confounding factors (such as childhood socioeconomic status, genetic predisposition) or reverse causality (such as pre-existing diseases may affect health behaviors and cognition). Nevertheless, after controlling for a series of important demographic and behavioral confounding factors, the pattern of education being associated with chronic disease risk through specific pathways revealed by this study still has significant public health implications, providing clear hypotheses and key directions for subsequent longitudinal or intervention studies.

## 5 Conclusion

In conclusion, this study elucidates the protective role of education against chronic diseases in rural China, operating through distinct cognitive, behavioral, and metabolic pathways. It highlights that strengthening health literacy and addressing key mediators like BMI are essential strategies. Future longitudinal research is needed to confirm causal pathways and evaluate the impact of specific educational and structural interventions on the chronic disease burden in transitioning rural communities.

## CRediT authorship contribution statement

**Tang Bin:** Supervision, Software, Methodology, Formal analysis. **Jia Xin:** Investigation, Formal analysis. **Zhu Siqi:** Software, Methodology, Investigation, Data curation. **Fu Meng:** Writing – original draft, Methodology, Formal analysis, Data curation. **G.U. Yang:** Writing – original draft, Methodology, Funding acquisition, Formal analysis, Data curation. **Rao Songli:** Writing – review & editing, Methodology, Investigation, Funding acquisition. **Zhang Ying:** Writing – review & editing, Software, Methodology, Investigation. **Lai Guihong:** Writing – original draft, Validation, Software, Resources. **Zhu Yuqing:** Writing – review & editing, Supervision, Project administration, Methodology, Conceptualization.

## Declaration of generative AI and AI-assisted technologies in the writing process

The author(s) declared no AI and AI-assisted technologies were used in the writing process.

## Funding

No funding was received.

## Declaration of competing interest

The author(s) declared no potential conflicts of interest.

## Data Availability

All processed data and models used during the study are available from the corresponding author by request.
